# The Distinct Roles of CXCR3 Variants and Their Ligands in the Tumor Microenvironment

**DOI:** 10.3390/cells8060613

**Published:** 2019-06-18

**Authors:** Nathan Reynders, Dayana Abboud, Alessandra Baragli, Muhammad Zaeem Noman, Bernard Rogister, Simone P. Niclou, Nikolaus Heveker, Bassam Janji, Julien Hanson, Martyna Szpakowska, Andy Chevigné

**Affiliations:** 1 Immuno-Pharmacology and Interactomics, Department of Infection and Immunity, Luxembourg Institute of Health (LIH), L-1526 Luxembourg, Luxembourg; nathan.reynders@lih.lu (N.R.); alexbar74@yahoo.it (A.B.); Martyna.Szpakowska@lih.lu (M.S.); 2 Faculty of Science, Technology and Communication, University of Luxembourg, L-1526 Luxembourg, Luxembourg; 3 Laboratory of Molecular Pharmacology, GIGA-Molecular Biology of Diseases, University of Liège, CHU, B-4000 Liège, Belgium; dabboud@uliege.be (D.A.); j.hanson@uliege.be (J.H.); 4 Laboratory of Experimental Cancer Research, Department of Oncology, Luxembourg Institute of Health (LIH), L-1526 Luxembourg, Luxembourg; muhammadzaeem.noman@lih.lu (M.Z.N.); bassam.janji@lih.lu (B.J.); 5 Laboratory of Nervous System Diseases and Therapy, GIGA-Neuroscience, University of Liège, B-4000 Liège, Belgium; bernard.rogister@uliege.be; 6 Neurology Department, CHU, Academic Hospital, University of Liège, B-4000 Liège, Belgium; 7 NorLux Neuro-Oncology Laboratory, Department of Oncology, Luxembourg Institute of Health (LIH), L-1526 Luxembourg, Luxembourg; Simone.Niclou@lih.lu; 8 Research Centre, Saint-Justine Hospital, University of Montreal, Montréal, QC H3T 1C5, Canada; nikolaus.heveker@recherche-ste-justine.qc.ca; 9 Department of Biochemistry, University of Montreal, Montréal, QC H3T 1J4, Canada; 10 Laboratory of Medicinal Chemistry, Center for Interdisciplinary Research on Medicine (CIRM), University of Liège, CHU, B-4000 Liège, Belgium

**Keywords:** chemokine receptor, CXCR3, CXCL11, CXCL10, CXCL9, CXCL4, tumor microenvironment, GPCR, ACKR3/CXCR7

## Abstract

First thought to orchestrate exclusively leukocyte trafficking, chemokines are now acknowledged for their multiple roles in the regulation of cell proliferation, differentiation, and survival. Dysregulation of their normal functions contributes to various pathologies, including inflammatory diseases and cancer. The two chemokine receptor 3 variants CXCR3-A and CXCR3-B, together with their cognate chemokines (CXCL11, CXCL10, CXCL9, CXCL4, and CXCL4L1), are involved in the control but also in the development of many tumors. CXCR3-A drives the infiltration of leukocytes to the tumor bed to modulate tumor progression (paracrine axis). Conversely, tumor-driven changes in the expression of the CXCR3 variants and their ligands promote cancer progression (autocrine axis). This review summarizes the anti- and pro-tumoral activities of the CXCR3 variants and their associated chemokines with a focus on the understanding of their distinct biological roles in the tumor microenvironment.

## 1. Introduction

Chemokines, or chemotactic cytokines, are small, secretory proteins that form the largest group within the cytokine family [1]. They bind to their cognate chemokine receptors, which belong to the G protein-coupled receptor (GPCR) superfamily, and mediate cellular responses as a result of downstream signaling pathways initiated by the activation of G proteins and regulated by the recruitment of arrestins [1,2,3]. To date, 48 chemokines and 23 receptors have been identified in humans. The chemokine–receptor network is highly intricate; a chemokine can bind one or many receptors, while a receptor usually recognizes several chemokines [1,4]. Moreover, a recent paradigm shift brought to light the concept known as biased signaling according to which GPCRs, including chemokine receptors, depending on the ligand or cellular context, trigger distinct signaling pathways [3,5,6]. Chemokines primarily arbitrate various immune responses through the migration of chemokine-receptor-expressing leukocytes to the target tissues, while they also orchestrate the development and maintenance of lymphoid or non-lymphoid tissues by inducing the migration, proliferation, and differentiation of stromal and immune cells [7,8,9,10,11,12]. In addition, chemokines play key roles in the tumor microenvironment (TME) as they are secreted by and act on the different cellular components comprising tumor cells, T- and B-lymphocytes, natural killer (NK) cells, macrophages, dendritic cells (DCs), fibroblasts, and vascular and lymphatic endothelial cells and their associated pericytes. Together with other soluble factors such as growth factors, inflammatory cytokines, and extracellular matrix enzymes, they contribute to cancer development and the formation of a cancer-specific immunity, influencing the maturation, activation, and trafficking of tumor-infiltrating immune cells [11,13,14,15,16,17,18,19].

**Figure 1:**
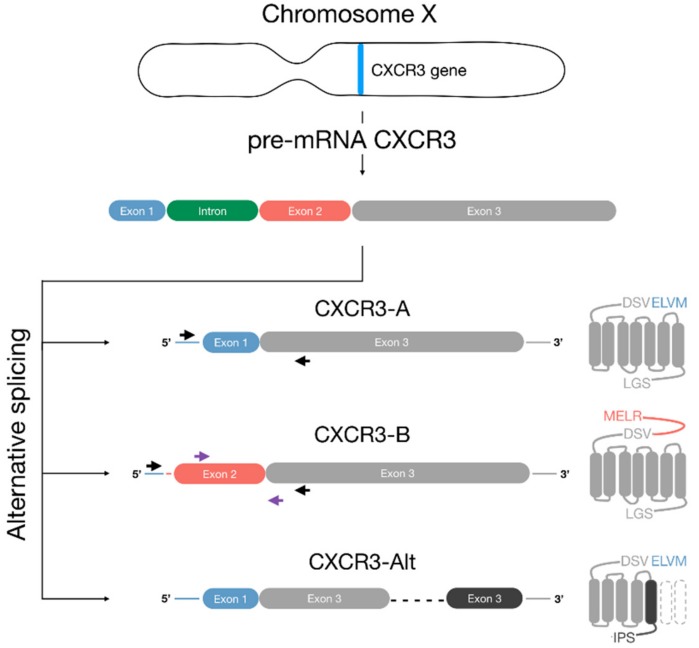
Schematic representation of CXCR3 variants. Due to alternative splicing of the pre-mRNA of the *CXCR3* gene, located in chromosome X, three CXCR3 variants can be generated. The CXCR3-A variant is the product of the splicing of the exon 1 and exon 3 within the *CXCR3* gene. The assembly of exon 2 and exon 3 results in the CXCR3-B variant, which has an N terminus longer by 52 amino acids (AAs) compared with CXCR3-A. The removal of the intron, exon 2, and a 337-bp region within the third exon during RNA splicing results in the CXCR3-Alt variant that comprises the N terminus and the first four transmembrane domains identical to CXCR3-A, as well as a possible fifth transmembrane region and a C terminus which are different from CXCR3-A and -B. The primers used to detect CXCR3-A, which also recognize CXCR3-B, and CXCR3-B are represented by the black and purple arrows, respectively.

The interferon (IFN)-gamma inducible chemokines CXCL11, CXCL10, and CXCL9, and the platelet-derived chemokines CXCL4 and its non-allelic variant CXCL4L1, exert their biological function by binding to the IFN-gamma inducible chemokine receptor CXCR3 [6,20,21,22]. Interestingly, due to alternative splicing of the CXCR3 mRNA, three variants have been reported, CXCR3-A, CXCR3-B, and CXCR3-Alt, which exhibit differences in their expression profiles, their N or C terminus, and the number of transmembrane domains (Figure 1). These structural differences alter the physiological characteristics of the receptors, including ligand-binding properties, signaling pathways, and cellular responses [23]. CXCR3-A and CXCR3-B are the two variants that have been best studied. They differ by a 52-amino acid (AA) extension at the N terminus of CXCR3-B and exert opposing cellular effects [24]. On the one hand, binding of CXCL11, CXCL10, and CXCL9 to the CXCR3-A variant induces chemotaxis and proliferation of cells by activating G_i_ and G_q_ proteins and triggering their downstream signaling pathways, including the intracellular Ca^2+^ release, ERK1/2, and Akt pathways [5,23,25,26,27,28,29,30,31,32,33,34]. The activation of CXCR3-A by CXCL4 and its variant CXCL4L1 remains to be elucidated [30,31,35]. On the other hand, stimulation of CXCR3-B by these chemokines inhibits cell migration and proliferation and induces apoptosis. The mechanism behind this opposite cellular response driven by CXCR3-B is still unclear; however, one study suggests a coupling to the G_s_ protein [24].

While CXCR3 and its endogenous ligands are mainly involved in inflammation and wound healing [8,36], they have also been described to have a dual role in tumor progression and immunity. This review aims to outline the impact of the CXCR3 ligand–receptor axis and its expression changes on the TME with a focus on the CXCR3-A and CXCR3-B variants.

## 2. The Crosstalk of the CXCR3 Variants and Their Chemokine Ligands Within the Tumor Microenvironment

Chemokines are abundantly present in the TME and play key roles in inducing proliferation of benign and malignant cells, leukocyte migration, and angiogenesis [11,12,37,38]. These processes can be initiated and maintained in paracrine and autocrine fashions.

### 2.1. CXCR3-A on Leukocytes Mediates their Migration to the TME to Modulate Tumor Progression

The presence of tumor-infiltrating leukocytes (TIL) in the TME is known to influence tumor development [39]. Following the discovery of CXCR3-A on activated T-lymphocytes [25] and the anti-tumoral activity of CXCL10 [40], the possibility of an anti-tumoral response through the migration of CXCR3-A+ leukocytes to the TME was proposed [41]. The importance of CXCR3-dependent anti-tumoral activity was confirmed by Hensbergen et al. where CXCL11-producing EL4 lymphoma cells, injected in mice, were rejected due to the infiltration of CXCR3+ CD8+ T lymphocytes and macrophages [42]. Similar observations were reported in murine models of renal cell carcinoma (RCC) (RENCA) and spontaneous melanoma (B16F10) where the reduced tumor growth resulted from an increased presence of CXCR3-A-expressing CD4+ and CD8+ lymphocytes and NK cells in the tumor bed [43,44,45]. In another study, melanoma (B16F10) or breast cancer (E0771) cells injected in CXCR3^−/−^ mice showed a significant increase in tumor growth compared to wild type (WT) mice, which was associated with a lower prevalence of CD8+ and CD4+ T cells as well as NK cells. This TIL-dependent anti-tumoral activity was furthermore validated in B16F10 tumor-bearing Rag2^−/−^ mice, which showed a significantly increased tumor growth when transferred with CXCR3^−/−^ cytotoxic T-lymphocytes (CTLs) compared to WT CTLs [46]. Such anti-tumoral activity of CXCR3-A+ leukocytes was also detected in human breast and gastric cancers as well as in RMA lymphoma [47,48,49]. Interestingly, the regressive characteristic of melanoma or certain melanocytic lesions was proposed to depend on the increased attraction of CXCR3-A+ cytotoxic lymphocytes to the TME [50].

In contrast, the higher prevalence of CXCR3+ regulatory T cells (Tregs) in human ovarian carcinomas was suggested to dampen the effector cell response, thus favoring the progression of the tumor [51]. This pro-tumoral effect of CXCR3+ Tregs was also observed in hepatocellular carcinoma (HCC), where a correlation could be made between CXCR3–CXCL10-dependent Treg infiltration and increased tumor growth and HCC recurrence after liver transplantation [52]. Moreover, in a chemically inducible murine model of skin carcinoma, CXCR3 knockout (KO) mice developed fewer tumors compared to WT mice. This observation was linked to a reduced presence of CXCR3-expressing CD3+ T cells, suggesting a cell proliferative effect on epidermal cells and an pro-tumoral activity of these TIL [53].

Taken together, these data suggest that the expression of CXCR3-A on leukocytes is needed to attract them to the TME and allows an anti-tumoral activity that diminishes tumor growth. However, the attraction of Tregs or other T-lymphocytes to the TME could also have a pro-tumoral effect by inducing cell proliferation and inhibiting the antitumor activity of effector leukocytes.

Of note, an adequate recruitment of CTLs in the tumor bed is not always observed. Correlated to a poor survival, it was for instance demonstrated that only 16% of patients with esophageal squamous cell carcinomas (ESCCs) or adenocarcinomas showed detectable CD8+ T cell infiltration within the tumor [54]. In fact, insufficient recruitment of activated T lymphocytes to the tumor bed is also recognized as one of the major hurdles in the current immunotherapeutic approaches [55]. Notably, the relevance of CXCR3-driven responses in immunotherapy has been demonstrated in several studies. In a B16-OVA mouse model, Mikucki et al. identified CXCR3-mediated trafficking at the tumor vasculature as important for effective T-cell-based cancer therapy by showing that CXCR3 is required for the extravasation of CTL to the tumor bed [45]. Similarly, in a B16 melanoma mouse model, the deletion of CXCR3 in CTLs lead to an impaired anti-tumoral response due to the failure of CTL infiltration in the TME upon anti-PD-1 treatment, suggesting CXCR3-dependent anti-PD-1 based anti-tumoral response [46]. Moreover, the blockade of PD-1 on naïve T cells enhanced CXCR3 expression and their absolute number in the spleen [56]. These studies show that CXCR3 is a potential target to increase the effectiveness of current immunotherapies.

### 2.2. CXCR3-A on Malignant Cells in the TME Contributes to Tumor Growth and Dissemination

As described above, the expression of CXCR3-A on leukocytes mediates their migration to the TME to exert their anti- and pro-tumoral activity; however, the CXCR3-A variant is also known to induce proliferation of various cell types, including human mesangial cells [24]. Therefore, the presence of CXCR3-A on tumor cells can induce an adverse effect by promoting and sustaining tumor development. Indeed, an increased proliferation of malignant glioma cells compared with normal astrocytes [57] and an anti-apoptotic effect in CXCR3-A-overexpressing human myeloma cell lines (HMCLs) was observed after CXCL10 treatment [58]. The increased expression of CXCR3-A on stage II colorectal cancer (CRC) and papillary thyroid cancer (PTC) cells was also associated with increased tumor development and negative prognosis [59,60]. In line with this, immunohistochemical analysis of localized clear cell RCC patient samples showed strong staining for CXCR3, which was correlated with a negative disease prognosis [61].

In addition to tumor development, expression of CXCR3-A on tumor cells promotes their dissemination. B16F10 melanoma injected into the footpads of mice showed macroscopic metastatic tumors in the lymph nodes already after one week, which could be attenuated with antisense CXCR3-A RNA [62]. An immunohistological analysis of primary invasive cutaneous melanoma samples from patients also supported the implication of CXCR3-A in tissue invasion and metastasis of malignant cells [63]. Furthermore, a metastatic potential conferred by CXCR3-A was observed in other human diseases, including epidermotropic and other B-cell disorders [64,65], glioblastoma (GBM) [66,67], CRC [68,69], high-grade serous ovarian cancer (HGSC) [70], lung adenocarcinoma [71] and breast cancer [72,73].

In conclusion, the expression of CXCR3-A on malignant cells leads to an increased proliferation and dissemination.

### 2.3. CXCR3-B Has an Anti-Proliferative Effect in the Tumor Microenvironment

In contrast to CXCR3-A, the CXCR3-B variant was shown to inhibit cell migration and proliferation [24]. CXCR3-B was first detected on human microvascular endothelial cells (HuMVECs) [24], but has also been found on CD4+ T lymphocytes [74], airway epithelial cells [75], and pericytes [76]. Although CXCR3-B is often expressed concomitantly with CXCR3-A, distinct CXCR3 variant expression levels can be observed on diverse cell types. Endothelial cells and pericytes abundantly express CXCR3-B [24,76], whereas CXCR3-A is not detectable [76,77], and activated T lymphocytes show higher CXCR3-A expression levels compared with CXCR3-B [31]. Various cancers evolve mechanisms to shift the CXCR3 variant expression levels towards ratios that are more beneficial for tumor maintenance or progression. For instance, human RCC downregulate CXCR3-B, which results in the upregulation of the anti-apoptotic heme oxygenase-1 (HO-1) and an increased tumor cell proliferation and dissemination [78,79]. Similarly, lower CXCR3-B mRNA levels were observed in ovarian and breast cancer cells compared with non-cancerous epithelial cells [80,81]. Moreover, decreasing the expression of CXCR3-B in breast cancer cells in vitro, using CXCR3-B-specific siRNA, resulted in increased cell proliferation and expression of anti-apoptotic proteins [81,82]. In addition to the inhibitory effect of CXCR3-B on cell proliferation, it has been observed that a rise in CXCR3-B expression could lead to increased tumor necrosis and lower the metastatic ability of tumor cells [83,84]. Of note, cancer stem cell-like properties upon CXCR3-B overexpression were reported in vitro in a breast cancer cell line, which could be prevented by silencing its mRNA [84].

Overall, it seems that cancer cells lower their expression of CXCR3-B to promote tumor proliferation, survival and dissemination and that this cellular effect can be reversed by enhancing CXCR3-B expression.

### 2.4. Is the CXCR3-B Variant Involved in Angiogenesis Regulation?

Tumors require adequate blood supply to obtain the sufficient oxygen, nutrients and other metabolic components to ensure their growth and survival. In contrast to normal angiogenesis, in which new blood vessels mature rapidly and show slow cell turn-over, tumor angiogenesis is an uncontrolled process where new blood vessels are formed in an irregular fashion [85,86]. Due to the high expression of CXCR3-B on endothelial cells and the angiostatic effect of CXCL10, CXCL9, and CXCL4 [24,87,88,89,90], it was proposed that the CXCR3-B variant could be responsible for this negative impact on tumor angiogenesis. Arenberg et al. indeed reported a decrease in angiogenesis and neovascularization in squamous cell carcinoma (SCC) after addition of CXCL10 in the TME that resulted in an attenuation in tumor growth and dissemination to the lung [91]. A similar observation was made following injection of CXCL9 to the tumor site in a mouse RENCA model [43]. In contrast, a rise in CXCL11 had no impact on the angiogenesis of mouse EL4 lymphoma cells nor human CRC [42,92]. Considering the expression of CXCR3 variants was not analyzed in these studies, one can only speculate that the inhibitory effect of these chemokines on tumor angiogenesis is driven through CXCR3-B present on endothelial cells, a phenomenon which may be cancer-type-dependent.

Although the above-mentioned data point to CXCR3-B-dependent angiostatic and inhibitory effects on cell proliferation, it has also been suggested that these are instead GAG-mediated. Indeed, both WT and CXCR3 KO endothelial cells showed an equivalent inhibitory effect on cell proliferation in the presence of CXCL10, which could not be reversed by neutralizing anti-CXCR3 antibodies. Moreover, the generation of a CXCL10 mutant devoid of CXCR3 signaling properties could also inhibit human umbilical cord endothelial cell (HUVEC) proliferation [93].

### 2.5. The CXCR3 Ligands are Upregulated in the TME and Act in an Autocrine and Paracrine Manner

Tumor cells as well as endothelial and immune cells secrete chemokines in the TME, which induce the recruitment of immune cells, modulate tumor immunity and promote tumor cell proliferation as well as metastasis [12]. Cancer cells also control the production and the activity of chemokines through the transcriptional silencing [94,95,96] or the post-translational modifications, via dipeptidyl peptidase 4 (DPP4 or CD26) [97,98]. These modifications undermine the attraction of tumor-invading lymphocytes, which impairs their anti-tumor effect [99,100] and the success of immunotherapy. In addition, CXCR3-endogenous ligands have been described to activate distinct signaling pathways, leading to specific cellular responses. For example, CXCL9 and CXCL10 induce effector Th1/Th17 cells and CXCL11 drives a Treg and Th2-biased effector cell polarization upon activation of CXCR3 on CD4+ T cells, while they upregulate tumor cell-related proteins that are beneficial for their survival, such as PD-L1 [21,101,102]. Here, we give an outline on the impact of the TME on the expression and secretion of CXCL11, CXCL10, CXCL9, CXCL4 and its variant CXCL4L1, and the effect of these chemokines on the different cellular components present within the TME.

#### 2.5.1. CXCL11

Although CXCL11 is the most potent CXCR3 ligand [103], its role in CXCR3-driven processes is sometimes overlooked due to its impaired production in certain mouse strains; such as in C57BL/6 mice where a 2-bp insertion after the start codon results in a premature stop codon and ultimately CXCL11 null mutant mice [101,104,105]. CXCL11 is also less available compared with CXCL9 and CXCL10, whether in health or disease state [106,107,108]. For CXCL11, being the full agonist of CXCR3 [109], its production may also be submitted to stricter regulation. In addition, ACKR3, formerly known as CXCR7, is a scavenger receptor for CXCL11 that may reduce the availability of CXCL11 in vivo and in the tumor bed. Indeed, ACKR3 has been shown to be upregulated on various tumor cells as well as on tumor-associated vasculature, therefore potentially interfering with the CXCR3–CXCL11 interactions [103,110,111,112].

Nevertheless, several studies pinpointed the importance of CXCL11–CXCR3 axis within the TME. It has been reported that mice challenged with EL4 T-cell lymphoma cells genetically modified to produce murine CXCL11 attracted more CD8+ lymphocytes and macrophages with anti-tumoral activity to the TME compared to WT EL4 T-cell lymphoma cells [42]. On the other hand, a study on human colon adenocarcinoma showed enhanced tumor growth and invasiveness after injection of CXCL11 to the TME [92]. It has also been described that the repression of CXCL11 in CRC tissues diminished the tumor cell growth and metastasis [113]. In addition, the observed increased expression of CXCL11 in HCC cells was linked with the upregulation of stem cell-related genes and the acquisition and maintenance of self-renewal and tumorigenic properties of tumor-initiating cells through an autocrine axis [114].

#### 2.5.2. CXCL10

Although CXCL10 is only a partial agonist of CXCR3, its impact on many human pathologies including cancer has been extensively studied. In glioma cells, for example, the expression of CXCL10 is significantly upregulated when compared with healthy astrocytes and has been shown to increase cell proliferation by acting on CXCR3 in an autocrine manner [57]. This pro-tumoral CXCL10 feed-forward loop was also observed in breast cancer; albeit, CXCL10 also attracted more CD4+ and CD8+ lymphocytes to the TME, in a paracrine manner, which attenuated tumor progression [115].

This pro- and anti-tumoral activity of CXCL10 could also be observed in CRC. Stage IV CRC patients showed an increased presence of CXCL10 in the TME, which was associated with a poor prognosis and promoted metastasis to the liver [116,117]. In contrast, higher secretion of CXCL10 in CRC increased the infiltration of CD8+ T lymphocytes and inhibited the ability of cancer cells to metastasize [118]. In addition, patients with ESCC showing higher CXCL10 expression had a better overall survival rate when compared with CXCL10-negative patients [119], while enhanced proliferation and metastatic behavior of melanoma tumor cells could be linked to higher CXCL10 secretion [120].

CXCL10 having opposing effects on TME that may occur simultaneously, the overall outcome of the disease seems to be tumor-type dependent and thus hard to predict.

#### 2.5.3. CXCL9

Similar to CXCL10, CXCL9 is one of the most extensively studied CXCR3 chemokines in cancer and also plays a dual role in the TME. CXCL9 gene upregulation in CRC and non-small-cell-lung-cancer (NSCLC) patient samples correlated with an increased overall survival, suggesting a beneficial impact of higher secretion of this chemokine in the TME [121,122]. In line with this observation, higher secretion of CXCL9 in HGSC and fibrosarcoma tumors also resulted in an increased overall survival presumable due to an enhanced attraction of leukocytes, in particular T and NK cells, to the tumor bed [95,123]. The anti-tumoral activity of CXCL9 could also be observed in lymphocyte-rich gastric carcinoma (GC) and regressing Burkitt’s lymphoma tumors. These regressing tumors showed a higher prevalence of CXCL9 in the TME compared with conventional tumors and exhibited an increased presence of TIL and tumor necrosis rate [124,125]. In contrast, it has been reported that the higher CXCL9 levels in melanoma—secreted by the melanoma endothelial cells—disrupt the endothelial barrier, which in turn leads to a higher dissemination of melanoma cells. This transendothelial migration of the tumor cells was diminished upon addition of anti-CXCL9 monoclonal antibody (mAb) [126]. The CXCL9-mediated metastasis was also observed in tongue squamous cell carcinoma (TSCC). TSCC cell show an increased CXCL9 and CXCR3 expression, which results in cytoskeleton alterations and decreased cell adhesion molecules that promotes tumor cell invasion and migration to the lymph nodes [127]. Interestingly, in follicular lymphoma (FL), elevated levels of CXCL9 result in lower event-free survival after treatment with immunotherapy implying a potential negative role of this chemokine after cancer treatment [128]. Thus, depending on the tumor cell type, CXCL9 can have either an adverse or a beneficial effect. This beneficial effect appears to rely on leukocyte attraction to the TME in a paracrine fashion; however, the adverse effect of CXCL9 on the malignant cell might not be direct as for CXCL10.

#### 2.5.4. CXCL4 and its CXCL4L1 Variant

While CXCL4 is mainly released after activation of platelets, it can also be secreted by stromal cells and tumor cells. In the two most frequent types of human lung adenocarcinomas (KRAS and TP53) and in CRC, an increase in CXCL4 expression was detected and correlated to lower patient survival probably due to its proliferative effect on tumor cells and anti-proliferative effect on CTLs [129,130,131]. The CXCL4L1 non-allelic variant has also been described to bind to CXCR3-A and -B [132]. The production of CXCL4L1 in melanoma, lung carcinoma and pancreatic adenocarcinoma (PDAC) inhibits tumor growth as well as neovascularization. Furthermore, CXCL4L1, as well as CXCL4, are able to recruit T cells, NK cells and DC to the TME, which may contribute to their anti-tumoral activity [132,133,134,135].

## 3. Technical Hurdles to Studying the CXCR3 Variants

The identification of the CXCR3-B variant, with its opposite cellular responses, raised new issues pertaining to the study and validation of the exact expression profile, signaling and the differential contribution of the two isoforms in CXCR3-mediated processes. Variant-specific tools were thus needed to be able to attribute the various cellular responses on the different cell types to CXCR3-A or CXCR3-B.

Nucleotide probes, or primers, were the first easily accessible tools to detect and distinguish endogenous expression of CXCR3-A- and CXCR3-B-specific transcripts in tissue and cells. The most frequently used primers, described by Lasagni et al., detect the spliced mRNA encoding both CXCR3-A and CXCR3-B as they bind to sequences that are common to the two variants. Nevertheless, the PCR products can be distinguished based on their different DNA band size. Primers designed for specific detection of CXCR3-A or CXCR3-B were also reported [24] (Figure 1). The CXCR3-A-specific primer aligns with the nucleotide sequence coding for the first seven amino acids present only in this variant [136] while the primer specific to CXCR3-B aligns within its extended N terminus. Besides PCR-based approaches, another application of nucleotide probes is in situ hybridization. This technique was used to screen for mRNA expression of the CXCR3 variants directly in slices of fixed tissues allowing the direct visualization of the receptor distribution and identification of receptor-positive cell types [137].

Variant-specific nucleotide probes proved to be easily manufactured and useful tools to evaluate the gene expression levels. However, confirming the presence of CXCR3 at protein level and, in particular, on the cell surface turned out to be a more difficult task. For the time being, the anti-CXCR3-A antibodies also recognize the CXCR3-B variant due to the usage of CXCR3-A N-terminus-derived peptides for immunization. The production of the first CXCR3-B-specific mAb raised against the extended 52-AA N terminus and its use in immunofluorescence and immunohistochemistry was first described by Lasagni et al. [24]. Meanwhile, other CXCR3-B-specific mAbs, using different CXCR3-B N-terminus-derived peptides as immunogen, are available but usually show an application spectrum limited to ELISA or Western Blot. To circumvent the staining of both CXCR3 isoforms, an alternative may be the use of conformational antibodies against each variant as previously generated for other GPCRs or chemokine receptors, including CXCR4 [138] CCR5 [139] and ACKR3 [109]. Indeed, the opposite cellular response of the two CXCR3 variants suggests that the presence of the N-terminal extension of CXCR3-B modifies the general tertiary structure of the receptor and might therefore expose different discontinued epitopes compared with CXCR3-A. Although their use would be limited to the detection of CXCR3 in its native conformation, it would help to confirm and quantify the presence of the two variants on the cell surface and understand the biology behind the selective receptor expression on the various cell types of the TME.

The diverse cellular effects of the CXCR3 variants were proposed to rely on the activation of different downstream signaling pathways. To investigate and identify CXCR3 variant-specific pathways, novel variant-selective modulators (antagonists, agonists or allosteric modulators) are needed as the currently used endogenous chemokines bind and activate both variants almost indifferently. Although CXCR3-A small-molecule modulators have been generated, their activity on CXCR3-B remains to be elucidated [106,140,141,142]. In addition, no CXCR3-B-specific small molecules have been reported so far. Therefore, the generation of small modulators is timely and would enable to unravel the CXCR3 variants signaling and could hold potential therapeutic value.

## 4. Conclusions, Challenges, and Future Directions

Over the last two decades, much effort has been made to understand the biology of the chemokine receptor CXCR3. Although the mechanism controlling the alternative splicing of both CXCR3 variants remains to be elucidated, numerous data on their signaling pathways and their respective contribution in tumor development and immunity have been generated.

On the one hand, it was shown that tumor cells can manipulate the expression of the CXCR3 variants in a way that is overall more beneficial for their maintenance. Indeed, by upregulating the expression of CXCR3-A and lowering the expression of CXCR3-B at their surface, tumor-initiating cells are able to enhance their proliferative potential and survival through autocrine stimulation loops that are further reinforced by the simultaneous upregulation of the expression of the CXCR3 ligands in the TME.

**Figure 2:**
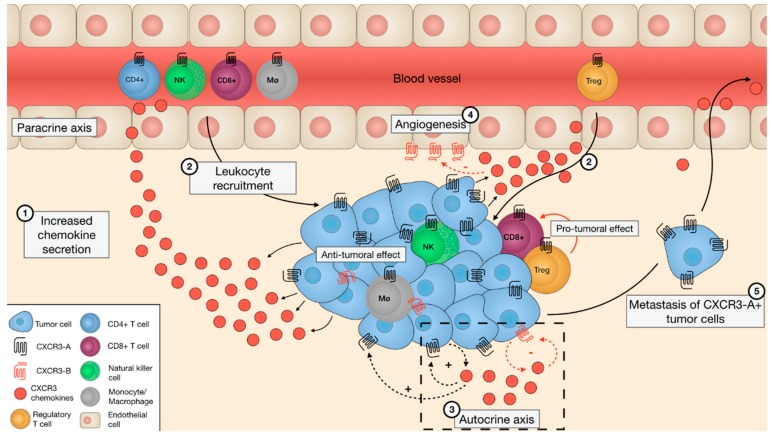
Illustration of the roles of CXCR3 variants and their ligands in the tumor microenvironment. The increased secretion of CXCR3 ligands in the TME, originating from the tumor cells, results in an increased chemokine gradient (1) and the recruitment of CXCR3-A+ leukocytes towards the tumor (paracrine axis) (2). The Th1-related immune cells drive anti-tumoral responses, while the recruited CXCR3-A+ Tregs have a pro-tumoral effect by suppressing other immune cells. The secreted chemokines also bind to CXCR3-A or CXCR3-B, present on secreting (autocrine axis) and neighboring tumor cells, and depending on the cellular context, may induce a proliferative or inhibitory effect (3). Moreover, the chemokines in close proximity to the endothelial cells attenuate the angiogenesis by activating CXCR3-B (4). In addition, the increased expression of CXCR3-A, together with decreased CXCR3-B expression, on the tumor cells enable them to detach from the tumor mass and disseminate to a distant tissue (5).

On the other hand, the increased secretion of CXCR3 ligands within the TME was shown to enhance the recruitment and the infiltration of various leukocytes into the tumor bed in a paracrine fashion, thereby modulating tumor progression. In a similar manner, chemokines participate in the control of metastasis of CXCR3-expressing tumor cells (summarized in Figure 2).

The demonstration of the impact of the CXCR3 axis in cancer points to its potential interest as a therapeutic target. However, the dual activity of CXCR3-A in the proliferative loops and the attraction of immune cells to the tumor bed, and the opposing roles of the CXCR3 variants’ ligand–receptor axis, as well as ligand and cell context bias, are important aspects that need to be considered for the development and use of anti-CXCR3 drugs or neutralizing antibodies. In addition, other players in the TME, including growth factors, inflammatory cytokines, and extracellular matrix enzymes, and the impact of the tumor on the microenvironment, such as induction of hypoxia, influence the CXCR3 axis and add yet another level of complexity to CXCR3-regulated processes, ultimately making the outcome of potential blockade hard to predict.

Therefore, a better understanding of all aspects of the biology of CXCR3 variants, including their exact signaling pathways and cellular responses in the tumor-microenvironment context, is urgently needed before envisaging the CXCR3 variants as potential anti-tumoral targets and the development of CXCR3 variant-specific drugs to be used for combined therapy, for example with anti-PD-1 immunotherapy, to turn cold to hot tumors.
